# Safety and Outcomes of Permanent and Retrievable Inferior Vena Cava Filters in the Oncology Population

**DOI:** 10.1155/2020/6582742

**Published:** 2020-02-05

**Authors:** Saba S. Shaikh, Suneel D. Kamath, Debashis Ghosh, Robert J. Lewandowski, Brandon J. McMahon

**Affiliations:** ^1^Northwestern University Feinberg School of Medicine, Department of Medicine, Chicago, IL 60611, USA; ^2^Northwestern University Feinberg School of Medicine, Department of Medicine, Division of Hematology/Oncology, USA; ^3^University of Colorado School of Public Health, Department of Biostatistics and Informatics, USA; ^4^Northwestern University Feinberg School of Medicine, Department of Radiology, Section of Interventional Radiology, USA; ^5^University of Colorado School of Medicine, Department of Medicine, Division of Hematology, USA

## Abstract

**Background:**

The role for inferior vena cava (IVC) filters in the oncology population is poorly defined.

**Objectives:**

Our primary endpoint was to determine the rate of filter placement in cancer patients without an absolute contraindication to anticoagulation and the rate of recurrent VTE after filter placement in both retrievable and permanent filter groups. *Patients*/

**Methods:**

A single-institution, retrospective study of patients with active malignancies and acute VTE who received a retrievable or permanent IVC filter between 2009-2013. Demographics and outcomes were confirmed on independent chart review. Cost data were obtained using Current Procedural Terminology (CPT) codes.

**Results:**

179 patients with retrievable filters and 207 patients with permanent filters were included. Contraindication to anticoagulation was the most cited reason for filter placement; however, only 76% of patients with retrievable filters and 69% of patients with permanent filters had an absolute contraindication to anticoagulation. 20% of patients with retrievable filters and 24% of patients with permanent filters had recurrent VTE. The median time from filter placement to death was 8.9 and 3.2 months in the retrievable and permanent filter groups, respectively. The total cost of retrievable filters and permanent filters was $2,883,389 and $3,722,688, respectively.

**Conclusions:**

The role for IVC filters in cancer patients remains unclear as recurrent VTE is common and time from filter placement to death is short. Filter placement is costly and has a clinically significant complication rate, especially for retrievable filters. More data from prospective, randomized trials are needed to determine the utility of IVC filters in cancer patients.

## 1. Introduction

Inferior vena cava (IVC) filters have become a common part of the management of venous thromboembolic (VTE) disease [1, 2]. Current guidelines recommend against the use of IVC filters in patients who can be anticoagulated [3]. However, widely used guidelines from the American College of Chest Physicians (ACCP) and the Society of Interventional Radiology (SIR) do not always concur in many other areas. Additionally, many filters are placed without a guideline-directed indication [4].

The role for IVC filters in the oncology population remains unclear. These patients have a two- to sixfold increased risk for VTE compared to the noncancer population. Furthermore, anticoagulation is often contraindicated either due to complications from the underlying disease or cancer therapy. As a result, IVC filters are frequently used despite limited data on their safety and efficacy in cancer patients [5]. Previously published studies have raised questions regarding the utility of IVC filter placement in patients with advanced-stage cancer given that they are more likely to die from progressive cancer rather than complications of VTE [6–9].

Current data indicates that cancer patients who can be anticoagulated do not benefit from IVC filters, similar to the general population. In a prospective study of 64 cancer patients with deep vein thrombosis (DVT) and/or pulmonary embolism (PE) receiving fondaparinux, patients randomized to IVC filter placement had no improvement in recurrent VTE rate or overall survival compared to those who without IVC filters [10].

For patients with relative contraindications to anticoagulation, IVC filters are frequently used in various clinical settings despite sparse randomized data on their utility in this context, especially in the cancer population. Clinical application of the data can be challenging given the variability in filter type, institutional expertise in filter placement, and heterogeneity of the patient populations receiving filters [11–13]. Traditionally, cancer patients were considered better candidates for permanent IVC filters given their ongoing high risk for complications with anticoagulation. However, the use of retrievable filters is increasing in this patient population [14, 15].

IVC filters have inherent risks and complications, including filter fracture, IVC perforation, embolization of filter fragments, and IVC occlusion. Retrievable filters have up to a sixfold increase in adverse events compared to permanent filters [14, 16]. Furthermore, there are substantial financial costs associated with both retrievable and permanent filters.

While there are guidelines available regarding indications for IVC filter placement, the degree to which they are utilized in clinical practice is unclear. We sought to evaluate provider practices regarding placement of IVC filters in a high-risk oncology patient population. Despite lack of data demonstrating clinical benefit, we hypothesize that IVC filters are commonly used in patients with a poor overall prognosis and often without a clear indication.

## 2. Materials and Methods

This was a single-institution, retrospective study of patients with active malignancies that had placement of either a retrievable or permanent IVC filter by interventional radiology between January 2009 and November 2013. Active malignancy was defined as a cancer requiring current treatment or at least consideration for active treatment within the last 6 months. Approval was obtained by the institutional review board of Northwestern University Feinberg School of Medicine.

All patients were logged into a database at the time of filter placement and were followed prospectively after filter placement. Additional data were obtained via independent retrospective review of patient charts. Baseline characteristics were recorded, including patient demographics, type and stage of malignancy, history of VTE, type of filter placed, and anticoagulation status after filter placement at discharge. Outcome measures included indication for filter placement, recurrent VTE rate, time between filter placement and death, and cause of death. Outcome measures specific to the retrievable filters group included rate of filter retrieval, time between filter placement and retrieval, and the rate of complications during filter retrieval.

Current Procedural Terminology (CPT) codes were used to obtain the cost of permanent filter placement and the cost of the permanent filter itself. CPT codes were also used to obtain the cost of retrievable filter placement and retrieval as well as the cost of the device itself.

Our primary objectives were to determine the rate of filter placement in cancer patients without an absolute contraindication to anticoagulation and to determine the rate of recurrent VTE after filter placement in both retrievable and permanent filter groups. Secondary objectives included survival after filter placement, cost of filter placement, and rates of retrieval and complications in the retrievable filter group.

### 2.1. Statistics

Final data analysis was performed for all included patients in each of the two study groups. Outcomes measured in the permanent and temporary filter groups were compared. Descriptive statistics were computed and presented in Tables 1–3. For binary variables, proportions were compared between the two groups using a two-sample test of proportions. For multicategorical variables, a chi-squared test of independence was used, and for continuous variables, a two-sample *t*-test was used to compare permanent and retrievable filters. Time from filter placement to death was compared for patients with stage IV cancer and plotted in Kaplan-Meier curves for the two groups. A log-rank statistic was used to test for equality of the survival distributions for the groups. All analyses were performed in R, and all inferences were performed at a significance level of 0.05.

## 3. Results

### 3.1. Patient Characteristics

After exclusions, 207 patients who underwent permanent filter placement and 179 patients who underwent retrievable filter placement between January 2009 and November 2013 were included. Five patients had retrievable filters placed twice. A summary of baseline patient characteristics is shown in Table 1. Patients who received a retrievable filter were more likely to have had a prior history of VTE (65% versus 40%, *p* = 0.00001). There was a significant association between filter group and TNM/Ann Arbor staging. Stage III/IV patients were more likely to have a permanent filter placed, whereas stage I/II or patients with hematologic malignancies were more likely to have a temporary filter placed.

**Figure 1:**
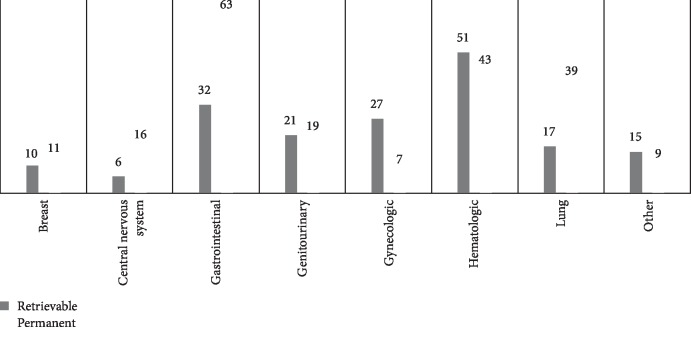
Number of patients who underwent filter placement categorized by cancer type.

In the retrievable filter group, filters were most commonly placed in patients with underlying hematologic malignancies (28%), GI malignancies (18%), and gynecologic malignancies (15%). In the permanent filter group, filters were most commonly placed in patients with GI malignancies (31%), hematologic malignancies (20%), and thoracic malignancies (19%) (Figure 1).

### 3.2. Filter Indications

A summary of reported reasons for filter placement is shown in Table 2. Contraindication to anticoagulation was the most cited reason for both the retrievable and permanent filter groups. 76% of patients in the retrievable filter group had a contraindication to anticoagulation cited as the indication for filter placement. Contraindications included upcoming procedures requiring temporary cessation of anticoagulation (32%), bleeding (30%), and thrombocytopenia (14%). Additional indications cited included failure of low-molecular-weight heparin (LMWH) (5%), concern for hemodynamic compromise from a PE (6%), and significant clot burden (7%). 165 patients (80%) in the permanent filter group had a contraindication to anticoagulation cited as the indication for filter placement. However, only 143 (69%) had an absolute contraindication, which included bleeding (42%), thrombocytopenia (19%), and upcoming or recent procedure (8%). 22 patients (11%) in the permanent filter group had either a relative contraindication to anticoagulation or a relative indication for filter placement. Six patients (3%) had the relative contraindication to anticoagulation of nonbleeding brain metastases. 16 patients (8%) had the relative indication for filter placement of recurrent VTE while on anticoagulation (i.e., failure of LMWH). There were no statistically significant differences between the groups regarding indications for filter placement.

42 patients (20%) with permanent filters had no relative or absolute contraindication to anticoagulation. Of these, filters were most commonly placed for IVC or lower extremity clot burden (33%) or poor cardiopulmonary reserve (24%). Importantly, these were previously considered relative indications for filter placement at some centers at the time of patient enrollment. Other cited indications in these 42 patients included fall risk, medication noncompliance, patient/family request, prophylaxis for anticipated immobility, gastric malignancy with concern for bleeding, nonspecific brain MRI findings, and discomfort from LMWH injections.

### 3.3. VTE Recurrence

There were a total of 87 recurrent thrombotic events following filter placement. In the retrievable filter group, 20% had recurrent VTE, 34% of which occurred on anticoagulation. Pulmonary emboli accounted for 21% of the recurrences despite having a filter in place, and lower extremity DVT accounted for the remaining 79%. In the permanent filter group, the rate of recurrent VTE was 24%, with 29% of recurrences occurring on anticoagulation and 33% of patients developing recurrent PE or IVC thrombus above the filter.

There were no statistical differences in the rate or type of recurrent VTE between the permanent and retrievable groups. Anticoagulation was more likely to be restarted prior to hospital discharge in those with a retrievable filter compared to permanent filter (63% versus 40%, *p* < 1 × 10^−4^).

### 3.4. Survival

By the end of the study, 102 (59%) patients had died in the retrievable filter group, most commonly due to progressive cancer. Of the patients that died, the median time from filter placement to death was 8.9 months. 83% of patients in the permanent filter group had died by the end of the study. The median time from filter placement to death was 3.2 months.

21 (10%) patients with permanent filters died within 10 days of filter placement. 86% of those patients had stage III or IV malignancies. The majority had either a GI malignancy (9 patients, 43%) or lung cancer (7 patients, 33%). In the permanent filter group, 33 (16%) patients died during the hospitalization in which the filter was placed.

**Figure 2:**
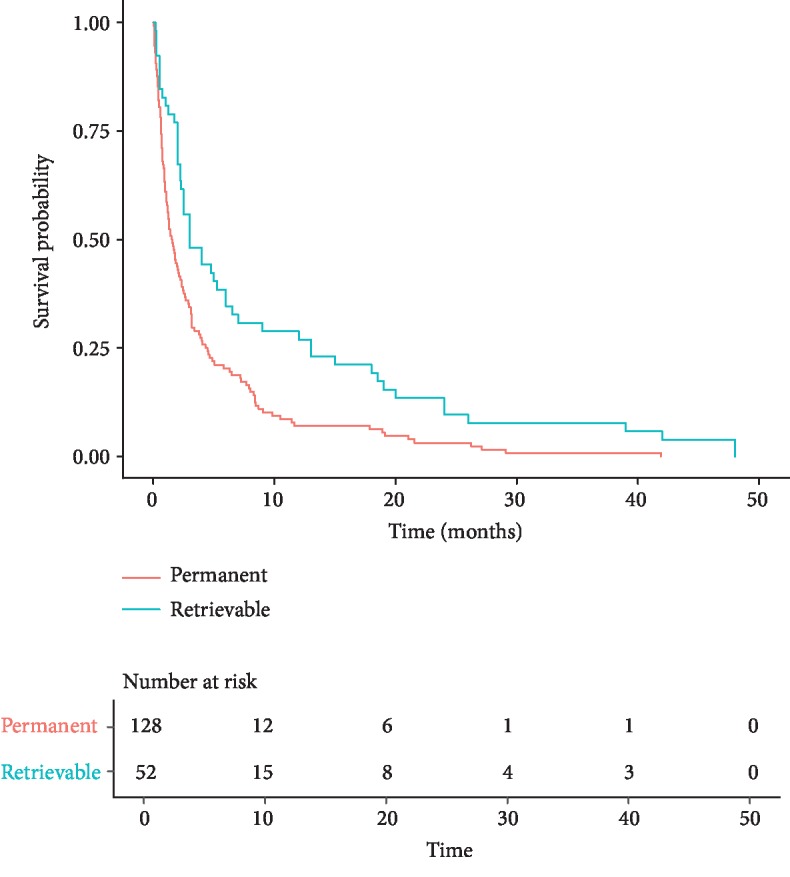
Survival for patients with stage IV cancer.

Stage of disease had a significant impact on survival. Kaplan-Meier curves for those with stage IV malignancies are shown in Figure 2. Patients with stage IV cancers with retrievable filters had a statistically significant higher probability of survival at 1 year compared to those with stage IV cancers and permanent filters (*p* = 0.00059). In the retrievable filter group, the median time to death from filter placement was 7 months in those with stage IV disease, with 84% alive at 1 month, 60% alive at 3 months, and 44% alive at 1 year. In the permanent filter group, 88% of the 146 patients with stage IV disease died with a median time to death of 1.9 months. 65% were alive at 1 month, 42% at 3 months, and 18% at 1 year.

Of the 157 patients with stage III or IV cancers in the permanent filter group, 34% had filters placed with either a relative or no contraindication to anticoagulation. Of these, 89% had died by the end of study with a median time to death from filter placement of 1.7 months.

Patients requiring intensive care and filter placement also tended to have poorer prognosis. Of the 36 (17%) patients who had permanent filters placed while in intensive care, 19 (53%) died during the hospitalization and an additional 12 (33%) had died by the end of study. The median time to death from filter placement for this subgroup was 0.6 months.

### 3.5. Retrieval and Complications

Of the 179 retrievable filters placed, 72 filters (40%) were retrieved, and 8 (11%) of those filters had abnormal findings documented at the time of filter retrieval. The most common abnormal finding was a clotted filter. 107 filters (60%) were not retrieved. These findings and reasons for filter nonretrieval are summarized in Table 3. For the filters that were retrieved, median time to retrieval was 1.35 months. Only 4% of patients had their filters retrieved during the same hospitalization.

### 3.6. Cost

Based on CPT codes, the cost of the retrievable filter itself was $1,576, placement was $10,983, and retrieval was $8,824 (total $21,383). The cost of a permanent filter was $4,695 and the cost of placement was $13,289 (total $17,984). The total cost of retrievable filters, retrievable filter placement and retrieval, with retrieval occurring in 40% of patients, was $2,883,389. The total cost of permanent filters and permanent filter placement was $3,722,688.

## 4. Discussion

This study demonstrates that IVC filters are frequently used in the oncology population despite limited prospective evidence for their efficacy and safety in this population. The Prévention du Risqué d'Embolie Pulmonaire par Interruption Cave (PREPIC) and PREPIC2 studies are the only prospective, randomized clinical trials assessing the utility of IVC filters that have been published to date. However, only 14% and 16%, respectively, of the patients in these trials had active cancers and both excluded patients not on anticoagulation [11–13]. Neither study showed a survival benefit for IVC filters in addition to anticoagulation, though the PREPIC study did show a reduced rate of recurrent PE with higher risk for recurrent lower extremity DVT. Those with cancer were at higher risk for recurrent VTE (HR = 2.46) and death (HR = 2.08). In a matched, case-control study of 32 cancer patients with VTE, half with IVC filters and half without, there were no differences in rate of recurrent VTE or survival between the two groups and there were no deaths related to VTE [7]. These results reinforce the intrinsic thrombotic nature of malignancy as well as the worse overall survival of these patients [13].

The risk-benefit analysis of IVC filter placement becomes particularly challenging in patients with end-stage cancer because life expectancy is limited in these cases. Survival after filter placement in our study was short and varied by filter group, with a median time from filter placement to death of 8.9 months in the retrievable filters group and 3.2 months in the permanent filter group. This was largely due to the advanced stage of the underlying malignancy in most cases and is consistent with prior series of cancer patients with VTE undergoing IVC filter placement. In a study by Wallace et al., patients with stage IV solid tumors had 76% survival at 1 month and just 29% at 1 year following filter placement [17]. Jarrett et al. noted more dismal statistics with a 6-week survival in stage IV patients following filter placement of 48% and a one-year survival rate of only 13.7% [18]. These findings suggest that the benefit of IVC filters for patients with end-stage cancer is not clear and may not justify the medical risks and costs.

Additional consideration must be made for the unique complications associated with retrievable filters and the rate at which these filters are retrieved. Available data suggest that retrieval rates are low, ranging from 11 to 46% [19–21]. At our institution, the interventional radiology department has a dedicated IVC filter clinic and an intensive follow-up strategy to monitor patient progress after filter placement and to contact referring providers at predesignated intervals regarding the appropriateness of filter retrieval. With this approach, retrieval rates have been reported to be as high as 60% [22]. Despite this dedicated approach, the rate of retrieval in this study for the cancer population was 40%. For most cases, this was attributed to clinician deferral in the setting of patient deterioration or continued contraindication to anticoagulation. While this is clinically reasonable, these complications were predictable at the time of filter placement in many cases and permanent filter placement or anticoagulation alone may have been more prudent.

Failure to retrieve filters may expose patients to additional risk as retrievable filters that are “converted to permanent” may have a greater risk for complications compared to permanent filters. In one study that surveyed the Manufacturer and User Facility Device Experience (MAUDE) database, 86.8% of the 1606 reported adverse events with IVC filters were related to retrievable filters and 13.2% to permanent filters [14]. Filter fracture was the most commonly reported event. Our study confirms a low retrieval rate despite having an intensive follow-up strategy. Earlier work by our group demonstrated cancer as a risk factor for failure to retrieve IVC filters [23]. This suggests that cancer patients that require an IVC filter should be offered a permanent device as retrievable filters are unlikely to be retrieved and may cause more complications [14].

Cost and financial toxicity are increasingly important considerations in the cancer population, especially in the final year of life. A 2010 study showed that costs in the final year of life ranged from $56,784 to $140,891, depending on the cancer type [24]. Other studies have illustrated the high out-of-pocket costs for cancer patients, which can exceed $100,000 [25–27]. Our study demonstrated a combined total cost of over $6.6 million in the 386 patients who underwent either permanent or retrievable filter placement. While this may be less than the cost of cancer-directed therapies, it is still a substantial cost for an invasive intervention with unclear benefits in the cancer population.

Our study has several limitations, including its retrospective, single-center design. These can introduce observation bias and limit the ability to generalize the data to other centers. Our study also lacks a matched control arm of cancer patients who did not receive IVC filters or of noncancer patients who did receive IVC filters. Nevertheless, this study is still significant in that it included a large number of patients with malignancy-associated thrombosis in both the retrievable and permanent filter groups.

## 5. Conclusions

The role for IVC filter placement in cancer patients remains unclear. Our results demonstrate that many patients with cancer have IVC filters placed without a clear indication, and their placement did not enhance outcomes, with high rates of recurrent VTE and poor overall survival. Prospective, randomized studies are needed to better delineate the indications for both permanent and retrievable IVC filters in the cancer population. IVC filters can contribute to the financial toxicity of cancer care and carry substantial risk for complications, especially with retrievable filters. Although there are circumstances in which a relative contraindication to anticoagulation may still be appropriate for filter placement, this should be considered on a case-by-case basis. When filter placement is still deemed necessary, a permanent filter should be strongly considered in the cancer population since retrieval rates are typically low. Additionally, our data demonstrate the need for better provider education regarding outcomes of patients with malignancy-associated thrombosis, indications for IVC filter placement in this population, and type of filter that is truly indicated. This may reduce interventions without clear benefit, particularly in patients with a poor overall prognosis.

## Figures and Tables

**Table 1:** Patient characteristics.

Characteristic	Retrievable IVC filters (*n* = 179)	Permanent IVC filters (*n* = 207)	*p* value
Median age (years)	64	65	
Male	85 (47%)	96 (46%)	0.91
Prior history of VTE	116 (65%)	83 (40%)	<0.00001
TNM or Ann Arbor staging			<0.0000001
Stage I or II	39 (22%)	9 (4%)	
Stage III or IV	89 (50%)	157 (76%)	
Hematologic malignancy	51 (28%)	41 (20%)	
Location of VTE			0.12
DVT	107 (60%)	100 (48%)	
PE	30 (17%)	41 (20%)	
DVT & PE	38 (21%)	62 (30%)	
IVC	4 (2%)	3 (1%)	
No VTE	0 (0%)	1 (0.5%)	
Anticoagulation on discharge			<0.0001
Started	113 (63%)	82 (40%)	
Not started	53 (30%)	92 (44%)	
Death before discharge	13 (7%)	33 (16%)	

*N*: number. Percentages of the total retrievable and permanent filter populations are indicated in parentheses.

**Table 2:** Indications for IVC filter placement.

	Retrievable IVC filters (*n* = 179)	Permanent IVC filters (*n* = 207)	*p* value
Contraindication to anticoagulation	136 (76%)	143 (69%)	0.16
Bleeding	53 (30%)	87 (42%)	
Thrombocytopenia	25 (14%)	39 (19%)	
Recent/upcoming procedure	58 (32%)	17 (8%)	
Relative contraindication to anticoagulation	9 (5%)	22 (11%)	0.99
Brain metastasis	0 (0%)	6 (3%)	
Failure of LMWH	9 (5%)	16 (8%)	
No contraindication to anticoagulation	34 (19%)	42 (20%)	0.85
IVC or lower extremity thrombus	13 (7%)	14 (7%)	
Pulmonary reserve	11 (6%)	10 (5%)	
Other	10 (6%)	18 (9%)	

Percentages of the total retrievable and permanent filter populations are indicated in parentheses. *N*: number; LMWH: low-molecular-weight heparin.

**Table 3:** Filter retrieval rates, complications, and reasons for lack of retrieval.

Filters retrieved	72
Filters with pathology documented at retrieval	8 (11%)
Clotted filter	5
Infection related to retrieval procedure	1
Thrombus noted after retrieval	1
Broken struts	1
Filters not retrieved	107
Reported reasons for not retrieving filters	
Progressive disease/clinical deterioration	55 (51%)
Continued inability to anticoagulated	25 (23%)
Lost to follow-up	8 (7%)
Inability to retrieve filter	5 (5%)
Recurrence with filter in place	4 (4%)
Concern for anticoagulation compliance	2 (2%)
Unknown	8 (7%)

Percentages for filters retrieved are out of 72 and percentages for filters not retrieved are out of 107.

## Data Availability

The data used to support the findings of this study are restricted in order to protect patient privacy. Data can be made available for researchers who meet criteria for access.
